# The complete mitochondrial genome of *Fejervarya kawamurai* (Anura: Dicroglossidae) and its phylogeny

**DOI:** 10.1080/23802359.2018.1467219

**Published:** 2018-04-28

**Authors:** Jian-Xiang Cheng, Yu-Ting Cai, Yu-Jie Zheng, Jia-Yong Zhang, Kenneth B. Storey, Yi-Xin Bao, Dan-Na Yu

**Affiliations:** a College of Chemistry and Life Science, Zhejiang Normal University, Jinhua, China;; b Key Lab of Wildlife Biotechnology, Conservation and Utilization of Zhejiang Province, Zhejiang Normal University, Jinhua, China;; c Department of Biology, Carleton University, Ottawa, Canada

**Keywords:** *Fejervarya kawamurai*, mitochondrial genome, Dicroglossidae, phylogeny

## Abstract

The mitochondrial genome of *Fejervarya kawamurai* is a circular molecule of 17,650 bp in length, containing 13 protein-coding genes, two rRNA genes, 23 tRNA genes (including an extra tRNA-Met), and the control region. The AT content of the whole genome is 56.9%. In Bayesian inference (BI) and Maximum likelihood (ML) analyses, we found that *F. kawamurai* is a sister clade to *F. multistriata* and *F. limnocharis*. The monophyly of *Fejervarya*, *Quasipaa*, *Nanorana* was well supported (1.00 in BI and 100% in ML).

*Fejervarya kawamurai* was described as a new species of dicroglossid frog and a member of the *Fejervarya limnocharis* complex from western Honshu, Japan mainland by Djong et al. (2011). It is distributed broadly across parts of Japan, Taiwan, and China (Frost 2018). There is much controversy about the classification of *F. kawamurai* and the closely related *F. limnocharis* and *F. multistriata* (Djong et al. 2011; Huang and Tu 2016). The complete mitochondrial genomes of *F. limnocharis* and *F. multistriata* were sequenced but the comparable genome of *F. kawamurai* was unknown. Hence, we sequenced the mitochondrial genome of *F. kawamurai* to discuss the relationship within the *Fejervarya limnocharis* complex.

Samples of *F. kawamurai* were collected from Zunyi city, Guizhou province, China (27°42′30′′, 106°55′12′). Whole genomic DNA was extracted from the hind leg muscle. The frog’s samples and DNA samples were stored at the College of Chemistry and Life Science, Zhejiang Normal University, China. DNA fragments were amplified using 15 pairs of highly conserved primers for mitochondrial genes which were designed according to the method of Liu et al. (2005) and Huang and Tu (2016). All PCR procedures were performed using an Arktik Thermal Cycler (Thermal Scientific,Shanghai, China). The PCR products were sequenced by Sangon Biotech Company (Shanghai, China).

The complete mitochondrial genome of *F. kawamurai* is 17,650 bp in length, containing 13 protein-coding genes, two rRNA genes, 23 tRNA genes (an extra tRNA-Met is present), and the control region. The AT content of the complete mtDNA is 56.9%. Most protein-coding genes begin with ATG as the start codon, except for ND5 gene with GTC, ND2 gene with ATT, COI gene with ATA, and ND3 gene with GTG. The ND6 gene is terminated with AGG as the stop codon, whereas ND1, ND2, ND5, COI, COII, ATP6, and COIII genes end with an incomplete stop codon (T–) and the other protein-coding genes end with TAA.

Bayesian inference (BI) and maximum likelihood (ML) trees were constructed using the 13 protein-coding genes of 25 species (Liu et al. 2005; Ren et al. 2009; Zhang et al. 2009; Zhou et al. 2009; Alam et al. 2010; Chen et al. 2011; Yu et al. 2012; Shan et al. 2014; Yu et al. 2015; Chen et al. 2015a,b; Jiang et al. 2016; Kiran et al. 2017), including *Occidozyga martensii* (Li et al. 2014) as outgroup to confirm the phylogenetic position of *F. kawamurai* in Dicroglossidae (Figure 1). To select conserved regions of the putative nucleotide sequences, each alignment was analyzed with the program Gblocks 0.91 b (Castresana 2000) using default settings. BI analysis and ML analysis was performed by MrBayes 3.1.2 (Huelsenbeck and Ronquist 2001) and RaxML HPC (Stamatakis et al. 2008), respectively. In both the BI and ML trees, we found that *F. kawamurai* is a sister clade to (*F. multistriata* +* F. limnocharis*). The monophyly of *Fejervarya*, *Quasipaa*, *Nanorana* was well supported (1.00 in BI and 100% in ML) as also reported in other recent studies (Zhang et al. 2009; Cai et al. 2018).

**Figure 1.**
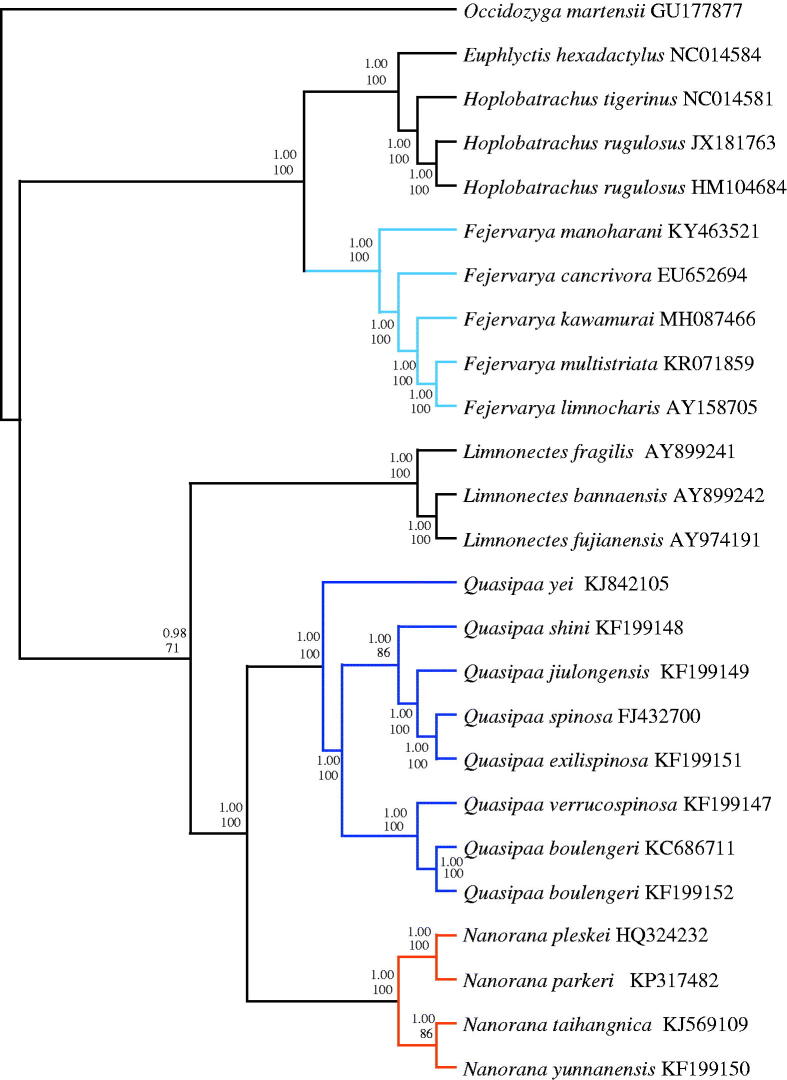
Phylogenetic relationships among 25 species of Dicroglossidae based on 13 protein-coding genes using nucleotide datasets. The tree was rooted with *Occidozyga martensii* as the out-group. Numbers above the nodes are the posterior probabilities of BI on top and the bootstrap values of ML on the bottom.

## Nucleotide sequence accession number

The complete mitochondrial genome of *F. kawamurai* has been assigned the GenBank accession number MH087466.

## References

[CIT0001] Alam MS, Kurabayashi A, Hayashi Y, Sano N, Khan MMR, Fujii T, Sumida M 2010 Complete mitochondrial genomes and novel gene rearrangements in two dicroglossid frogs, *Hoplobatrachus tigerinus* and *Euphlyctis hexadactylus*, from Bangladesh. Genes Genet Syst. 85:219–232. 21041980 10.1266/ggs.85.219

[CIT0002] Castresana J 2000 Selection of conserved blocks from multiple alignments for their use in phylogenetic analysis. Mol Biol Evol. 17:540–552. 10742046 10.1093/oxfordjournals.molbev.a026334

[CIT0003] Cai YT, Ma L, Xu CJ, Li P, Zhang JY, Storey KB, Yu DN 2018 The complete mitochondrial genome of the hybrid of *Hoplobatrachus chinensis* (*♀*) × *H. rugulosus* (*♂*) and its phylogeny. Mitochondrial DNA B. 3:344–345. 10.1080/23802359.2018.1450661 PMC7799566 33474164

[CIT0004] Chen G, Wang B, Liu J, Xie F, Jiang JP 2011 Complete mitochondrial genome of *Nanorana pleskei* (Amphibia: Anura: Dicroglossidae) and evolutionary characteristics of the amphibian mitochondrial genomes. Cur Zool. 57:785–805.

[CIT0005] Chen Z, Zhai X, Zhang J, Chen X 2015a The complete mitochondrial genome of *Feirana taihangnica* (Anura: Dicroglossidae). Mitochondrial DNA. 26:485–486. 24730606 10.3109/19401736.2014.908362

[CIT0006] Chen Z, Zhai X, Zhu Y, Chen X 2015b Complete mitochondrial genome of the Ye’s spiny-vented frog *Yerana yei* (Anura: Dicroglossidae). Mitochondrial DNA. 26:489–490. 24960568 10.3109/19401736.2014.926542

[CIT0007] Djong HT, Matsui M, Kuramoto M, Nishioka M, Sumida M 2011 A new species of the *Fejervarya limnocharis* complex from Japan (Anura, Dicroglossidae). Zool Sci. 28:922–929. 10.2108/zsj.28.922 22132790

[CIT0008] Frost DR 2018 Amphibian Species of the World: an Online Reference. American Museum of Natural History, New York, USA. Accessible at http://research.amnh.org/herpetology/amphibia/index.html [accessed 1 March 2018].

[CIT0009] Huang ZH, Tu FY 2016 Mitogenome of *Fejervarya multistriata*: a novel gene arrangement and its evolutionary implications. Genet Mol Res. 15:1–9. 10.4238/gmr.15038302 27706580

[CIT0010] Huelsenbeck JP, Ronquist F 2001 MrBayes: Bayesian inference of phylogenetic trees. Bioinformatics. 17:754–755. 11524383 10.1093/bioinformatics/17.8.754

[CIT0011] Jiang L, Ruan Q, Chen W 2016 The complete mitochondrial genome sequence of the Xizang Plateau frog, *Nanorana parkeri* (Anura: Dicroglossidae). Mitochondrial DNA Part A. 27:3184–3185. 10.3109/19401736.2015.1007327 25758045

[CIT0012] Kiran SK, Anoop VS, Sivakumar KC, Dinesh R, Mano JP, Kaushik D, Sanil G 2017 An additional record of *Fejervarya manoharani* Garg and Biju from the Western Ghats with a description of its complete mitochondrial genome. Zootaxa. 4277:491. 30308627 10.11646/zootaxa.4277.4.2

[CIT0013] Li E, Li X, Wu X, Feng G, Zhang M, Shi H, Wang L, Jiang J 2014 Complete nucleotide sequence and gene rearrangement of the mitochondrial genome of *Occidozyga martensii*. J Genet. 93:631–641. 25572222 10.1007/s12041-014-0418-4

[CIT0014] Liu ZQ, Wang YQ, Su B 2005 The mitochondrial genome organization of the rice frog, *Fejervarya limnocharis* (Amphibia: Anura): a new gene order in the vertebrate mtDNA. Gene. 346:145–151. 15716031 10.1016/j.gene.2004.10.013

[CIT0015] Ren Z, Zhu B, Ma E, Wen J, Tu T, Cao Y, Hasegawa M, Zhong Y 2009 Complete nucleotide sequence and gene arrangement of the mitochondrial genome of the crab-eating frog *Fejervarya cancrivora* and evolutionary implications. Gene. 441:148–155. 18848608 10.1016/j.gene.2008.09.010

[CIT0016] Shan X, Xia Y, Zheng YC, Zou FD, Zeng XM 2014 The complete mitochondrial genome of *Quasipaa boulengeri* (Anura: Dicroglossidae). Mitochondrial DNA. 25:83–84. 23841602 10.3109/19401736.2013.782023

[CIT0017] Stamatakis A, Hoover P, Rougemont J 2008 A rapid bootstrap algorithm for the RAxML web servers. Syst Biol. 57:758–771. 18853362 10.1080/10635150802429642

[CIT0018] Yu DN, Zhang JY, Li P, Zheng RQ, Shao C 2015 Do cryptic species exist in *Hoplobatrachus rugulosus*? An examination using four nuclear genes, the Cyt b gene and the complete MT genome. PLoS One. 10:e0124825. 25875761 10.1371/journal.pone.0124825 PMC4395372

[CIT0019] Yu DN, Zhang JY, Zheng RQ, Shao C 2012 The complete mitochondrial genome of *Hoplobatrachus rugulosus* (Anura: Dicroglossidae). Mitochondrial DNA. 23:336–337. 22708866 10.3109/19401736.2012.690748

[CIT0020] Zhang JF, Nie LW, Wang Y, Hu LL 2009 The complete mitochondrial genome of the large-headed frog, *Limnonectes bannaensis* (Amphibia: Anura), and a novel gene organization in the vertebrate mtDNA. Gene. 442:119–127. 19397958 10.1016/j.gene.2009.04.018

[CIT0021] Zhou Y, Zhang JY, Zheng RQ, Yu BG, Yang G 2009 Complete nucleotide sequence and gene organization of the mitochondrial genome of *Paa spinosa* (Anura: Ranoidae). Gene. 447:86–96. 19631263 10.1016/j.gene.2009.07.009

